# Predicting Ecological Roles in the Rhizosphere Using Metabolome and Transportome Modeling

**DOI:** 10.1371/journal.pone.0132837

**Published:** 2015-09-02

**Authors:** Peter E. Larsen, Frank R. Collart, Yang Dai

**Affiliations:** 1 Argonne National Laboratory, Biosciences Division, Argonne, IL, United States of America; 2 University of Illinois at Chicago, Department of Bioengineering, Chicago, IL, United States of America; University of Massachusetts, UNITED STATES

## Abstract

The ability to obtain complete genome sequences from bacteria in environmental samples, such as soil samples from the rhizosphere, has highlighted the microbial diversity and complexity of environmental communities. However, new algorithms to analyze genome sequence information in the context of community structure are needed to enhance our understanding of the specific ecological roles of these organisms in soil environments. We present a machine learning approach using sequenced Pseudomonad genomes coupled with outputs of metabolic and transportomic computational models for identifying the most predictive molecular mechanisms indicative of a Pseudomonad’s ecological role in the rhizosphere: a biofilm, biocontrol agent, promoter of plant growth, or plant pathogen. Computational predictions of ecological niche were highly accurate overall with models trained on transportomic model output being the most accurate (Leave One Out Validation F-scores between 0.82 and 0.89). The strongest predictive molecular mechanism features for rhizosphere ecological niche overlap with many previously reported analyses of Pseudomonad interactions in the rhizosphere, suggesting that this approach successfully informs a system-scale level understanding of how Pseudomonads sense and interact with their environments. The observation that an organism’s transportome is highly predictive of its ecological niche is a novel discovery and may have implications in our understanding microbial ecology. The framework developed here can be generalized to the analysis of any bacteria across a wide range of environments and ecological niches making this approach a powerful tool for providing insights into functional predictions from bacterial genomic data.

## Introduction

Terrestrial plants rarely exist simply as solitary organisms. Rather they encompass complex interacting communities of soil fungi, subsurface bacteria, and animals whose combined functions are crucial to above and below ground plant biomass [1, 2]. Some of these subsurface communities are correspondingly dependent upon their plant host. Around 20% to 40% of photosynthetically-derived sugars from plants are consumed directly by the subsurface community [3, 4], making the root-associated ecosystem an important part of the terrestrial carbon cycle. These communities reside and interact in the narrow region in the soil directly influenced by plant root exudates called the rhizosphere, and within the rhizosphere, soil bacteria fill multiple ecological niches. A niche is defined here the set of specific roles by which organisms interact with the abiotic environment, the plant roots, and other microbes as they compete for available nutrients.

Efforts to understand the compositions and interrelationships of bacteria in the rhizosphere community [1, 5–7] are limited by the inability to culture these organisms in the laboratory [8]. As such, much of the information about these organisms can only be learned indirectly (i.e. inferred from genomic sequences assembled from metagenomic data sets). In this context, ecological functions of uncharacterized but genomically sequenced bacteria are frequently inferred from genomic sequence homology to characterized species [9, 10]. This approach is not without limitations, however, since high levels of sequence homology between bacteria can occur between organisms of different ecological functions [9, 11–13]. Other approaches focus on the presence of key genes linked to ecological function, such as those for the fixation of nitrogen [14–16] or for injecting toxins into a host cell [17–20]. While a few important ecological characteristics can be inferred in this fashion, many ecological functions cannot be reliably linked to small sets of specific genes. More sophisticated approaches use computational approaches such as flux balance analysis (FBA) modeling of predicted bacterial metabolomes to infer a bacterium’s ecological niche [21, 22]. Although, FBA can infer a bacterium’s nutritional requirements, the approach is often not predictive for a bacterium’s role it its community.

To circumvent these limitations, we evaluated the utility of machine learning computational tools to infer a bacterium’s ecological role from genomic data. Support Vector Machine (SVM) models were used to predict rhizosphere ecological niches using outputs from system-scale computational models for genomic, metabolomic, and transportomic features. Given a set of training examples, each marked as belonging to one of two categories, such as membership to an ecological niche, an SVM training algorithm builds a model that assigns new examples into one category or the other. SVMs are particularly powerful in their ability to avoid over-fitting. To evaluate the utility of this computational framework, we selected Pseudomonads, a genus of bacteria commonly found in the rhizosphere community and of particular interest to terrestrial carbon cycling. Pseudomonads are widely distributed and sequence data from representative organisms indicate their genomes encode a diverse spectrum transporters, enzymes and secondary metabolic activities [23]. This functional and metabolic diversity makes these bacteria highly relevant to computational modeling of metabolic and transportomic capacities.

Pseudomonads occupy a wide variety of habitats including soil and marine environments and can be plant or animal pathogens [24, 25]. For the present analysis, the selections of environmental niche labels are based upon those previously reported by Silby et al [25] as well as other investigators (Identified in Table 1). Classes of ecological niches are non-exclusionary and a single Pseudomonad species may be associated with any number of niches. For the present analysis, we considered four ecological niches from the rhizosphere: biocontrol, biofilm formation, plant growth promotion, and plant pathogen. Biocontrol is an ecological niche associated with Pseudomonads in the rhizosphere in which the bacteria protect the plant’s roots from detrimental fungi, bacteria, or other pathogens [26–28]. Biofilm formation is the ability to form biofilms in any environment, but can include soils or bacteria which reside internal to the host organism [29–31]. Plant pathogenicity is the ability to cause disease in plant roots or leaves [32, 33]. Plant growth promotion is the ability to form beneficial relationships with plant roots that result in increased plant biomass [34]. For associating these ecological niches with molecular mechanisms, four modeling output types were considered: enzyme function profiles, metabolic models, secondary metabolism models, and transporter profile (transportomic) models. Enzyme function profiles are generated using the number and distribution of the genome encoded set of specific enzyme activities as identified by assignment of an Enzyme Commission (EC) annotation number. Similarly, transportomic models are generated using the transporter functions encoded in a set of bacterial genomes. Enzyme function abundances and a set of all possible metabolic transformations performed by those functions were used to derive the metabolic models predicting the relative rates of metabolic turnover for specific metabolites, and the transportomic models, predicting the relative capacity of bacteria to transport specific ligands across cell membranes. Secondary metabolism models were derived using subset of enzymes involved in the generation of secondary metabolites (organic compounds that are not directly involved in the normal growth, development, or reproduction of an organism).

**Table 1:** Assigned Ecological Niche Classifications of Pseudomonad Species.

Species	# Genomes	Biocontrol	Biofilm	Plant Pathogen	Plant Growth	Reference
Aeruginoa	9	N	Y	Y	Y	(Silby, Winstanley et al. 2011)
Brassicacearum	1	Y	N	N	N	(Ortet, Barakat et al. 2011)
Denitrificans	1	Y	N	N	N	(Ainala, Somasundar et al. 2013)
Entomophila	1	Y	N	N	N	(Vodovar, Vallenet et al. 2006)
Flourescens	4	Y	Y	Y	Y	(Silby, Winstanley et al. 2011)
Fulva	1	N	N	N	N	(Renault, Deniel et al. 2007)
Mendocina	2	Y	Y	N	N	(Silby, Winstanley et al. 2011)
ND	1	N	N	Y	N	(Li, Zhao et al. 2013)
Poae	1	Y	Y	N	Y	(Muller, Zachow et al. 2013)
Protogens	2	Y	Y	Y	Y	(Jousset, Schuldes et al. 2014)
Putida	11	Y	Y	N	Y	(Silby, Winstanley et al. 2011)
Stutzeri	6	N	N	N	Y	(Silby, Winstanley et al. 2011)
Syringae	3	Y	Y	Y	N	(Silby, Winstanley et al. 2011)

## Materials and Methods

### Pseudomonad Genomes

There were 43 fully sequenced and annotated Pseudomonad strains available from the NCBI (ftp://ftp.ncbi.nih.gov/genomes/) that are confidently associated with specific rhizosphere ecological niche classes at the time this analysis originated. The files for predicted protein sequences (.faa files in NCBI genomic sequence database) were used for all analysis strains. Pseudomonad rhizosphere ecological niche was defined as a function of Pseudomonad species and assigned based on published manuscripts (**Table 1**). The complete list of strains and accompanying references is available in **S1 Data**.

### Metabolomic and Transportomic Modeling

**Fig 1:**
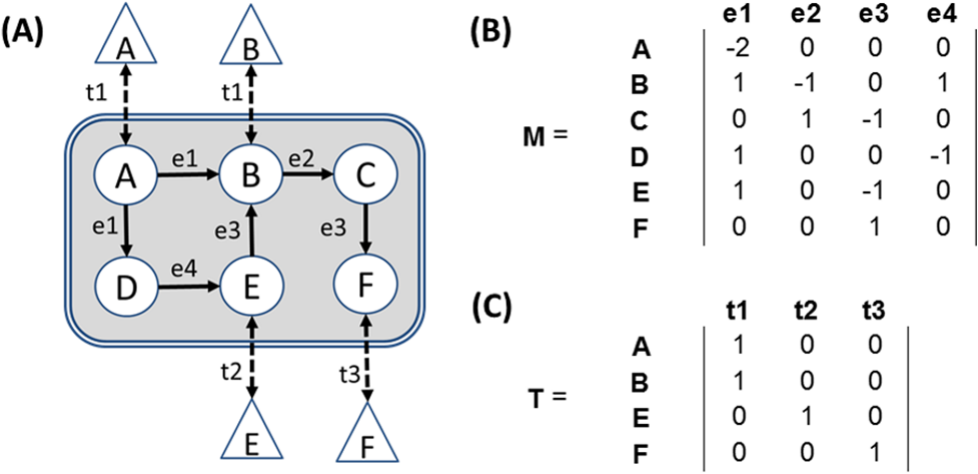
Metabolomic and Transportomic Modeling. In (A), a simplified metabolomic/transportomic network is featured. Triangles are extracellular compounds, circles are intracellular compounds, and double-line is cellular membrane. Dashed edges t1-t3 are transmembrane transport interactions. Solid arrows e1-e4 are directed metabolic transformations. Each enzyme or transporter annotation can be associated with one or more compounds. (B-C) represents the network in (A) transformed in matrices for use in PRMT and PRTT calculations. In (B), matrix does not consider enzymatic flux or mass balanced reactions. In (C), the transportome matrix is constructed such that a ‘0’ indicates that a ligand is not transported and a ‘1’ indicates that a ligand is transported by a transporter of a given annotation. For example, in the cartoon above, ligands A and B are transported by a transporter annotated with function ‘t1’.

SVMs were trained on the outputs of four different computational models which are generated using annotation data derived from sequenced and annotated genomic information: enzyme function profiles, metabolomic, secondary metabolism, and transportomic. The generation of each type is described below and the relationships between data types are pictured in **Fig 1**.

### Enzyme Function Profiles

All Pseudomonad predicted gene models from the published genomic sequence were re-annotated for protein functions. This approach ensured that all functional assignments for the predicted proteins from genomic sequence data use uniform annotation criteria and a consistent ontology for enzyme functions and ligands.

The database of the Kyoto Encyclopedia of Genes and Genomes (KEGG) was used as the source of annotated protein sequences of metabolic enzymes and transmembrane transporter activities [35, 36]. For enzyme function annotations, Enzyme Commission (EC) annotation numbers [37] were used. A database of bacterial enzymes annotated with EC numbers and associated with specific reactions in KEGG metabolic pathways (downloaded May 16, 2011) was used for this analysis. The set of 754,066 protein sequences is annotated with 2,605 unique EC number enzyme function descriptions and the complete collection of annotated enzymes is available, in FASTA-format in **S2 Data**. For transmembrane transporter function KEGG Orthology (KO) annotations were used [38]. The complete list of transmembrane transporter KO annotations used can be found in **S3 Data**. There are 164,321 protein sequences, annotated with 891 unique transporter/sensor functions, and are associated with the transport of 272 unique ligands in the set of annotated transmembrane transporters and the complete FASTA-formatted set of annotated transporter proteins is available in **S4 Data**. It is possible for a single protein sequence to be present in both the set of enzymes and the set of transmembrane transporters. Protein annotations were assigned to single best BLAST-P hit with e-values < 1x10^-10^ (NCBI-Blast 2.2.23+). Enzyme function profiles for Pseudomonads were generated as lists of all possible enzyme or transmembrane transporter annotations and the number of genes in each Pseudomonad for the assigned function.

### Metabolomic and Secondary Metabolism Models

Predicted Relative Metabolic Turnover (PRMT) uses enzyme function profiles for quantifying the relative metabolic turnover between two metabolomes and has been described in detail elsewhere [39]. The necessary tools for performing PRMT, instructions, and demonstration data can be downloaded from www.bio.anl.gov/PRMT.html and the computational approach is briefly summarized below.

Required input for PRMT is a set of relative unique enzyme function abundances and a set of all possible metabolic transformations performed by those functions (**Fig 1A**). EC annotations and KEGG metabolic pathways are used for this purpose. Enzyme function abundances are provided as vectors of the log_2_-transformed number of enzyme function representation in genomes of length *ec*, where *ec* is the number of enzyme function annotations in the metabolic model. The network of possible enzyme-mediated metabolic transformations is provided by a matrix ***M*** of size *m* by *ec*, where *m* is the total number of metabolites present in the metabolic network (**Fig 1B**). This matrix is the Enzyme Interaction Network (EIN) described in [39] and is generated using the PRMT script “GenerateEIN_fromECList.pl” (www.bio.anl.gov/PRMT.html).

The PRMT vector between metabolomes encoded by genomes *x*, and *y* is given by:

$$\vec{PRMT}\;=M\left(\vec{e^{x}}-\vec{e^{y}}\right)$$ (1)

The resulting vector of PRMT-scores of length *m* contains the comparison of predicted relative metabolic turnover of each metabolite in ***M*** for metabolome encoded by genome *x* relative to genome *y*. A positive PRMT score indicates an increased relative capacity for the synthesis of a compound in the metabolome encoded by genome *x* relative to genome *y*. A negative PRMT score indicates an increased relative capacity for the consumption of a compound in the metabolome encoded by genome *x* relative to genome *y*. PRMT scores do not indicate rates of reaction or predict quantities or concentrations of compounds in a metabolome. PRMT scores are generated using the script “CalculatePRMT_AllCols.pl” (www.bio.anl.gov/PRMT.html).

Two sets of PRMT models were generated. The first used the complete set of enzyme functions identified in the set of the 43 Pseudomonad genomes to generate the metabolomic models. For generation of secondary metabolism models, the set was restricted to the subset of enzyme activities that is present in the KEGG Biosynthesis of Secondary Metabolites pathway (KEGG map 01110). Both sets were calculated using the average enzyme function count across all Pseudomonads. In this analysis, the reference genome *y* is always calculated as the average unique enzyme function counts of all Pseudomonad genomes, as has been similarly done for normalization in previous applications of PRMT (e.g. [39–42]). Average unique enzyme function counts are calculated:

$${EFC}_{AVE}^{x}\;=\frac{\sum_{t=1}^{\text{max}\;t}\;{ECF}_{t}^{x}}{T},$$ (2)

where ${EFC}_{AVE}^{x}$ is the average enzyme function count for enzyme activity *x*, and ${EFC}_{t}^{x}$ is the enzyme function count for activity *x* in taxa *t* of a total of *T* taxa.

### Transportomic Models

Predicted Relative Transmembrane Transport (PRTT) is a system-scale metric that quantifies relative ability of organism to transport specific metabolites across the cellular membrane and is introduced here for the first time. PRTT-scores are calculated as a special case of PRMT-scores, using the same tools as PRMT, but using the pre-calculated matrix of transporter annotations and transported ligands as the EIN matrix. The transportomic matrix is available as **S5 Data** and for download from the PRMT website at www.bio.anl.gov/PRMT.html.

Required input for PRTT is the set of transporter function abundances and a matrix of transporter annotations and transported ligands. A selected subset of KO annotations was used for transporter annotations. Log_2_-transformed representations of transmembrane transport function annotations in genomes are provided as vectors of length *ko*, where *ko* is the number transporter function annotations in the transportomic model. Also required is a transporter ligand specificity matrix ***T*** of size *l* by *ko*, where *l* is the total number of ligands present in the transporter ligand specificity matrix (**Fig 1C**).

The PRMT score vector between transportomes encoded by genomes *x*, and *y* is given by:

$$\vec{PRTT}\;=\;T\left(\vec{k^{x}}-\;\vec{k^{y}}\right)$$ (3)

The resulting vector of PRTT-scores is of length *l* for the comparison of predicted relative transmembrane transport of each ligand in ***T*** for transportome encoded by genome *x* relative to genome *y*. A positive PRTT score indicates an increased relative capacity for transmembrane transport of a specific ligand in the transportome in genome x relative to genome *y*. A negative PRTT score indicates a decreased relative capacity of transmembrane transport of a ligand. PRTT scores do not indicate absolute rates or directionality of transmembrane transport activity. As with PRMT scores, all PRTT scores were calculated using reference genome *y* calculated as the average transmembrane transport function counts for all Pseudomonad genomes.

### SVMs and Training Procedure

SVMs to predict Pseudomonad ecological niche were trained using subsets of calculated enzyme profiles, metabolic and secondary metabolomic model outputs, and transportomic model outputs. Enzyme function profiles (**S6 Data**), PRMT scores (**S7 Data**), secondary metabolism PRMT scores (**S8 Data**), or PRTT (**S9 Data**) scores used as features in training SVMs were non-zero in more than half of the genomes and had a standard deviation greater than 0.2 indicating features were present in most Pseudomonas genomes and there is variation in feature values.

**Fig 2:**
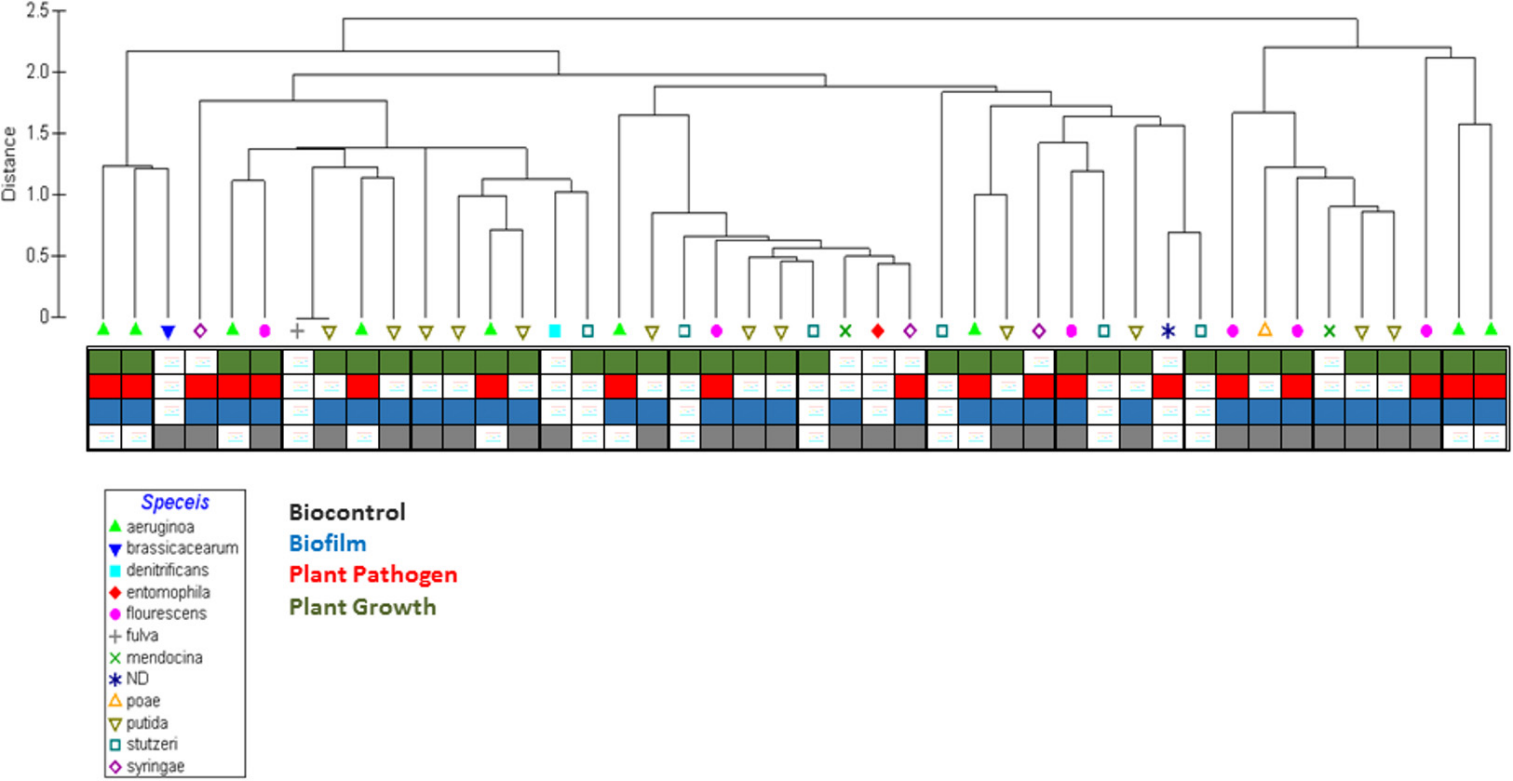
Clustering Pseudomonad genomes using enzyme function counts. The “Primer 6” core package and enzyme function profile data were used to generate hierarchical clusters. No obvious pattern by species or by ecological function is apparent using only enzyme function count and hierarchical clustering. Suggesting additional data and/or alternate methods are required to deduce Pseudomonad environmental niche using sequenced and annotated genomes.

SVMs were generated using a One Versus Rest (OVR) strategy, implemented as a set of four independent binary classifiers, and validated using a Leave One Out Validation (LOOV) scheme (Fig 2). In the OVR SVM binary classification approach, separate SVMs were generated for each ecological niche class (Biocontrol, Biofilm, Plant Pathogen, and Plant Growth Promotor), that is, Biocontrol vs non-Biocontrol, Biofilm vs. non-Biofilm, Plant Pathogen vs. non-Plant Pathogen, and Plant Growth Promoter vs. non-Plant Growth Promotor. A LOOV scheme is a special case of a *K*-fold cross validation. It is most appropriate for the data in this study as the number of Pseudomonas is small relative to the number of possible model features and some Pseudomonads are represented by a very small number of examples that would go un-represented in the training sets of a *K*-fold cross validation. In the LOOV experimental design, a single genome is used as a validation set and the model is trained on the remaining genomes with a 10-fold cross-validation procedure and linear kernels. The selection of validation sample and training SVM is repeated until each of the 43 Pseudomonas genomes was used as the validation sample once. For generation of SVM, package ‘e1071’ v1.6–1 in R-project (August 29, 2013, http://cran.r-project.org/web/packages/e1071/index.html) was used. The outputs collected included class predictions, decision values for all training and validation samples and SVM files. A total of 16 SVM models, each with 43 LOOV, were generated: Four feature types based on computational model output types (enzyme function profiles, metabolomic model, secondary metabolism model, and transportomic model) were used to train for the prediction for each of the four ecological niche classes (biofilm formation, biocontrol agent, plant pathogen, and plant growth promoter).

A prediction confidence measurement was assigned to class predictions of validation samples. Using the SVM model for the positive examples in SVM training sets, the averages and standard deviations of distances from the hyperplane of SVM classifier were calculated. The statistical significance of the prediction of validation sample was calculated using its SVM distance *x* from the SVM classifier hyperplane and the standard normal distribution:

$$\text{Confidence of prediction}\;=\;1-\int_{0}^{x}\frac{1}{\sqrt{2\pi }}e^{\left(-\frac{z^{2}}{2}\right)}$$ (4)

where *z*, the normalized distance for the validation sample, is calculated as *x* divided by the standard deviation of the absolute values of training set positive decision value distances. A confidence of greater than or equal to 95% was considered a significant assignment of validation sample to ecological niche class. The complete set of confidence values for all validation predictions can be found in **S1 Data**.

To quantitate the predictive power of SVMs, F-score was used. F-score is a metric that combines precision and recall of predictions and is calculated as follows:

$$F\;=\;2\;\times \;\frac{Precision\times Recall}{Precision+Recall}$$ (5)

where,

$$Precision=\;\frac{tp}{tp+fp}\;,\;Recall=\;\frac{tp}{tp+fn}$$ (6,7)

In *Precision* and *Recall*, *tp* is the number of true positives, *fp* is the number of false positives, and *fn* is the number of false negatives in predictions.

## Results

### Computational Model Overview

Enzyme function profiles were generated from the re-annotated Pseudomonad genomes. These profiles identified 1092 unique enzyme activities and 195 transmembrane transport annotations that were present in at least one genome. 606 of the enzyme functions were present and showed variation across Pseudomonads in over half of the re-annotated genomes and were used to train Enzyme Function Profile SVMs. Metabolic (PRMT-scores) and transportomic (PRTT-scores) models for Pseudomonads were calculated using the complete enzyme function profiles. The complete metabolomic model is comprised of 6642 enzymatic transformation interactions between 3688 metabolites, of which 2143 were present and showed variation across Pseudomonads in over half of the re-annotated genomes and were used to train metabolomic SVMs. The secondary metabolism model is comprised of 1649 enzymatic transformation interactions between 1494 metabolites, of which 714 are variable across Pseudomonads and were used to train secondary metabolism SVMs. The transportomic model is predicted to transport 271 metabolites, of which 169 are predicted to be variably transported and were used to train transportomic SVMs.

### Clustering by Enzyme Function Profiles Does Not Distinguish Between Ecological Niches

To determine if genomic data alone are sufficient to predict ecological niches, enzyme function profile data was used to generate hierarchical clusters using ‘Primer 6’ v6.1.10 (Primer-E Ltd., Lutton, UK). Hierarchical clustering of genomic representation of enzyme functions (**Fig 2**) shows that Pseudomonads do not group by species or by ecological niche annotation. This inability to cluster genomes into species ecological activity groups by hierarchical clustering with these data indicates that other computational approaches are required to predict ecotype from genomic information.

### SVMs Accurately Predict Rhizosphere Ecological Niche from System-Scale Model Outputs

**Fig 3:**
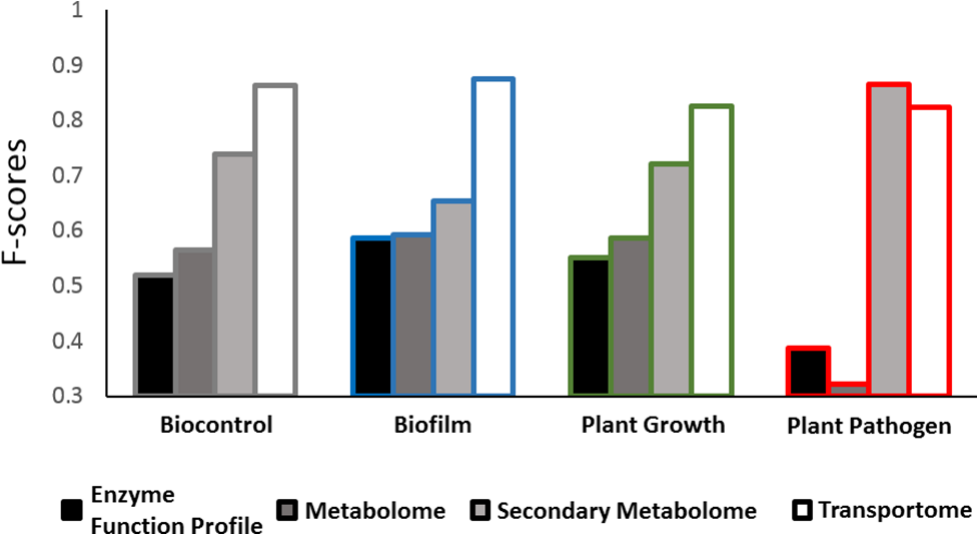
F-score for SVM predictions of ecological niche by input data type. Predictive capacity of SVM models is function of input type used to train model (enzyme profile, metabolomic, secondary metabolism or transportomic) and ecological niche in the rhizosphere (biocontrol, biofilm, plant growth promoter, or plant pathogen). Environmental niche classes were assigned at a confidence of > = 95%. F-scores are calculated from Leave One Out Validation (LOOV).

Accuracy of SVM predictions, as quantitated by F-score, varies by type of model output used to train SVMs and by niche type (**Fig 3**). The transportomic model was the most predictive (i.e. highest F-score) for three out of four environmental niches: biocontrol, biofilm, and plant growth. Secondary metabolism was most predictive for the plant pathogen ecological niche. Enzyme function profile and complete metabolomic model were never the most predictive for any environmental niche. Considering the average F-score by SVM classifier type, both secondary metabolism features (t-test p-value 0.009) and transportomic model features (p-value 0.0003) are significantly more predictive than enzyme function profiles. Average F-score for SVM prediction using transportomic model features was significantly higher than average for SVM trained with metabolomic model features (p-value 0.002).

To avoid consideration of redundancy between features in the secondary metabolism and the less predictive complete metabolome feature types, only enzyme function profile, secondary metabolism model, and transportomic model are considered in the subsequent analysis of high-weight SVM features.

### Highly Predictive SVM Features Provide Insights into Mechanisms of Adaptations to Ecological Niches

In SVMs, features used for training are assigned weights, proportional to their predictive capabilities with high-weight features more predictive than low weight features. We considered high-weight as more than 2 standard deviations +/- average feature weight for each SVM input type. The complete lists of high-weighted features are found in **Tables A-D** in **S2 File**. The biological relevance of many of these features is supported by prior published observations and discussed in subsequent sections.

**Fig 4:**
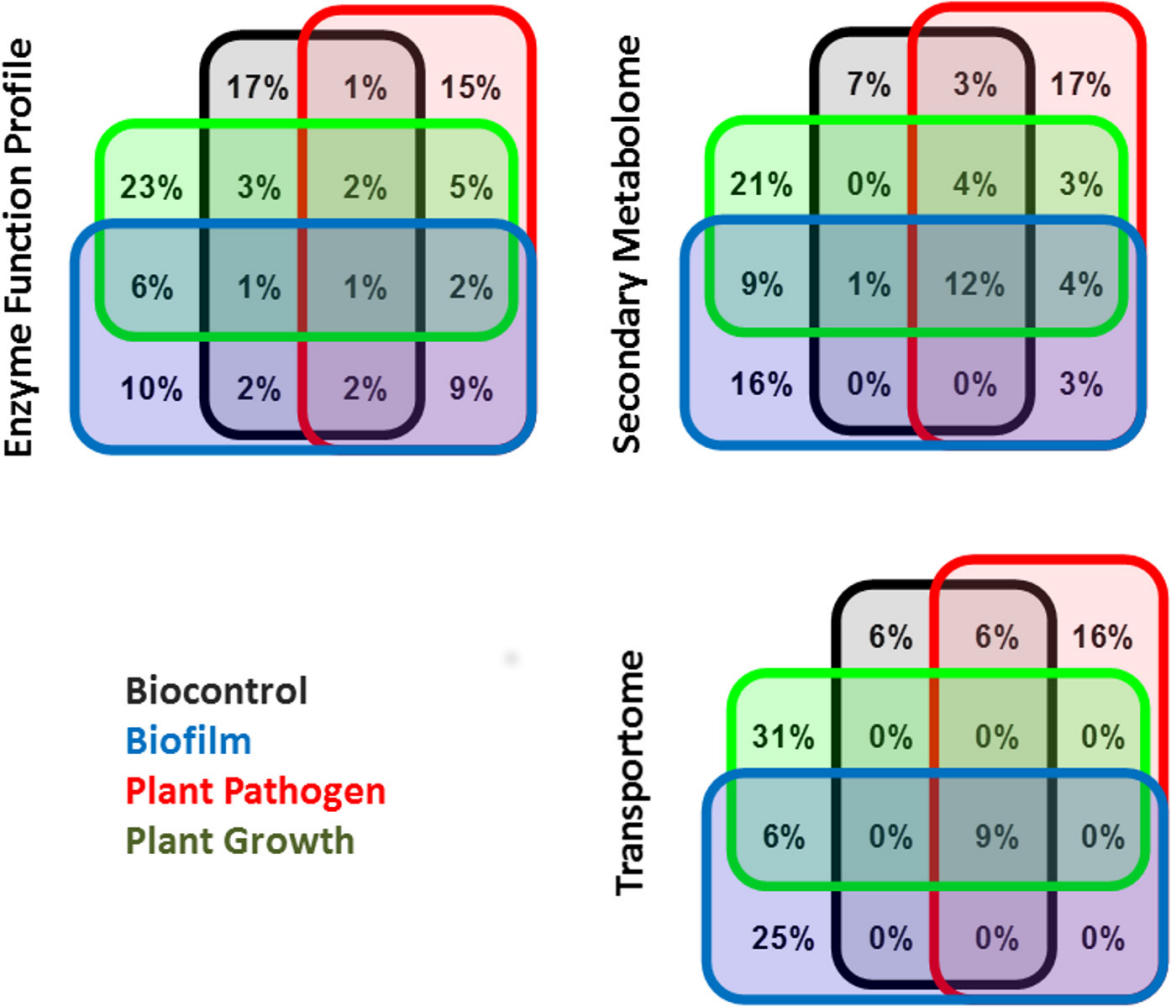
Venn diagram for significant features identified by SVM for each model feature type and for each ecological niche. All values in diagram are presented as percent of features out of total number of high-weight features for SVM feature type.

Highly predictive features for one ecological niche type are often present in the highly predictive features of another niche (**Fig 4**). For all training model output types, plant growth is the niche with the least overlap with other niches. Secondary metabolism has the largest proportion of high-weight features in common with all rhizosphere ecological niches. The transportomic model has the least overlap of high-weight features between niches with a small number of transported ligands common to all ecological niches.

### Rhizosphere

High-weight features that are common to all rhizosphere ecological niches **(Fig 4**) are the molecular signatures for Pseudomonads that occupy the rhizosphere ecosystem, relative to those Pseudomonads that inhabit other environments (**Tables A-D in S2 File**). Secondary metabolism and transportomic have the largest sets of rhizosphere-specific features and many of the functions associated with these features are consistent with growth characteristics of organisms in a soil environment. Transport activities that are identified as predictive for inhabiting the rhizosphere involve carbohydrate transporters (e.g. 2-O-alpha-manosyl-D-glycerate) suggestive for osmoregulation in soils [43, 44] and 3-hydroxyphenylpropionic, one of many lignin breakdown products, which are ubiquitous in soils. We also identified the general class of cation transport as predictive for inhabiting the rhizosphere. This transporter class is possibly associated with maintaining charge balance in negatively charged soils in Pseudomonads and other soil bacteria [45, 46]. The ability of rhizosphere Pseudomonads to import lignin breakdown products is particularly relevant for the known important saprophytic capacities of Pseudomonads [47] which consume organic matter in soils. Another key metabolomic predictor is the capacity for catecholamine biosynthesis (**Table C in S2 File**), which are regulatory compounds found in many plants that are involved in growth and development and are regulated by stress conditions [48].

### Biocontrol

Biocontrol is most predictive by its transportome (**Table D in S2 File**), specifically by transport of cobamide coenzyme (biochemically active forms of vitamin B12) and monosaccharides. Cobamide coenzyme is part of a vitamin biosynthesis pathway that induces resistance against pathogens and synthesis of growth factors in plant roots. Monosaccharide transport is a part of a sensor system associated with wound response and pathogen detection [49, 50]. Metabolomics (**Table C in S2 File**) predicts that metabolism of acetyl-D-glucosamine, a sugar that does not occur in plants or prokaryotes but is a structural polymer of fungi, is an indicator of biocontrol activity, specifically against fungal infection [51]. Additionally, intermediates in the pathway of isoniazid metabolism, which is an antimicrobial compound [52], is also identified as important to Pseudomonas biocontrol activities.

### Biofilm

The most predictive metabolic activities for biofilm formation (**Table C in S2 File**) is the metabolism of anti-biofilm compounds protoporphyrin [53, 54] and methyglyoxal [55, 56], suggesting that a metabolic feature of these organisms involves a defense against biofilm inhibition synthesized by other competitors in the rhizosphere. Additional pathways previously implicated in biofilm formation include antranilate degradation pathways implicated in biofilm formation in *P*. *aeruginosa* [57] and the shikimate pathway [58, 59], which was identified as predictive for biofilm formation. Important transport functions predictive for biofilm formation (**Table D in S2 File**) are growth required environmental nutrients, specifically phosphorus and nitrogen. Limiting availability of both of these nutrients is an inducer of biofilm formation [60–62].

### Plant Pathogen

Fatty acid biosynthesis pathways were identified as features predictive for plant pathogenicity in Pseudomonads. This computational prediction corresponds to the recently reported biological observation that lipid signaling is important for plant resistance to pathogens [63, 64]. Transport of plant sugars, such as arabinose [65, 66], and polyamines [67] are both important signals in plant stresses and defense against pathogens, and are also predictive of Pseudomonads’ pathogenicity.

### Plant Growth

Metabolomic input type (**Table C in S2 File**) predicts that synthesis of a number of plant signaling compounds is predictive of plant growth promotion by Pseudomonads including indole [68] and flavones eriodictyol, neringenin [69–71]. A number of transport functions were also identified (**Table D in S2 File**). C4-dicarboxylate is indicative of increased organic acid metabolism in the rhizosphere. Calcium transport is important in plant-root symbiote signaling [72, 73]. Glutathione transport is also predictive of plant growth promotion, and bacterially synthesized glutathione is previously reported as detected in the rhizosphere. Transport of a number of simple sugars (i.e. malonate, mannose, sucrose, galactose, and hexose) was found to be predictive of plant growth promotion by Pseudomonads and is suggestive of an ecological niche that is able to take advantage of exuded photosynthetic sugars present in the rhizosphere.

## Discussion

Our analysis indicated that SVMs trained on outputs from enzyme function profiles, metabolic models, and transportomic models can be used to accurately predict the ecological niche of biofilms, biocontrol, plant growth promotion, or plant pathogen for a group of Pseudomonad organisms. A simple hierarchical clustering based on enzyme function profiles failed to distinguish Pseudomonads at the level of species or by rhizosphere ecological niche, suggesting a need for alternate approaches. The accuracy of SVM prediction was dependent upon model feature types used to train the SVM, with enzyme function profiles being the least predictive (F-scores between 0.44 and 0.60) and transportomic models the most predictive (F-scores between 0.82 and 0.89). Of intermediate predictive power is metabolic modeling, with secondary metabolome model features being more predictive of ecological niche than those of the complete metabolome. These results suggest that the most characteristic capability of an organism to fit into an ecological niche is not the set of enzyme functions available to it, but the system-scale mechanisms by which the bacterium senses and interacts with its environment. The most predictive model feature type for biofilm formation, plant growth promotion, and biocontrol in Pseudomonas was identified at its transportome. Our analysis revealed a novel aspect that the capability of an organism to occupy an ecological niche is most indicated by its ability to sense and manipulate its environment via its transmembrane transport capacity. The ability to model and quantitate bacterial transportomes using PRTT may provide new insights into microbial ecology and evolution of function.

Analysis of the most predictive features, i.e. those features with the highest SVM weights, for each ecotype identifies considerable overlap with prior biological knowledge, suggesting that not only are metabolomic and transportomic model features highly predictive for ecological niche, but also return results that are biologically significant. The agreement between computational predictions and previously published observations indicates that this analysis framework yields a number of potential hypotheses suitable for the design of molecular biological experiments. While transportomic feature data are found to be most predictive alone, it is very possible that a model using mixed feature data types may prove to have higher overall predictive capabilities in future models. However, as optimizing predictive capacity, particularly on this relatively small set of Pseudomonad genomes, was not the goal of this research effort, we have elected not to present a mixed data-type SVM model here. In the context of a more general tool for accurately predicting ecological niches of uncharacterized bacteria from genomic data, such a mixed data-type SVM would be more appropriate and we are currently pursuing this goal.

Examining the most predictive features that were derived from each type of computational model data type provides a framework for a system-scale understanding of how specific molecular mechanisms in Pseudomonads contribute to their capacity to fill their varied ecological niche spaces in the rhizosphere. While Pseudomonads were considered here, the framework presented can be generalized for mining metagenomics and genomics data for new insights to bacterial functional determination and ecological niche prediction. As technology for sequencing and genome assembly continuously improves, the ability to generate completely sequenced bacterial genomes from environmental [74], clinical [75], or even single cell isolates [76] is expanding at an exponential rate. Of the 2749 completely sequenced and annotated genomes since 1995 listed in KEGG Organisms (http://www.genome.jp/kegg/catalog/org_list.html), over half have been generated since 2011. Many more thousands of draft bacterial genomes are currently in the process of completion and annotation. Yet only a relative handful of these bacteria have been characterized in the laboratory and the totality of what is known about many of these organisms is inferred from their genomic sequences. While the model generated in this study is not likely applicable to other taxa, the proposed framework can be applied to many other bacterial groupings and environmental niches. Computational approaches, such as the one that we have demonstrated here will be increasingly important to analyze and understand the role that bacteria play across all ecosystems.

## Supporting Information

S1 Data Compilation of Pseudomonas Species and Reference Data. (XLSX)

S2 Data FASTA-formatted file of 754,066 bacterial proteins annotated with EC enzyme functions. (7Z)

S3 Data List of KEGG Orthology (KO) annotations (TXT)

S4 Data FASTA-formatted file of 164,321 bacteria proteins annotated with KO transporter functions. (ZIP)

S5 Data Transporter-Ligand matrix. Matrix for use with PRMT scripts to generate PRTT-scores from tabular formatted data of transporter annotation counts in Pseudomonad annotated genomes.
(TXT)

S6 Data All Enzyme function profiles for 43 re-annotated Pseudomonads. This file is comprised of two lists: EC enzyme annotation function counts and KO transporter function counts.
(TXT)

S7 Data PRMT-scores for Pseudomonad metabolic models. (TXT)

S8 Data PRMT-scores for Pseudomonad secondary metabolic models. (TXT)

S9 Data PRTT-scores for Pseudomonads transportomic models. (TXT)

S1 File Ecological niche predictions for all Pseudomonads. There is one table in file for each feature type: **Table A**–Enzyme function profile, **Table B**- Metabolome, **Table C**–Secondary metabolome, and **Table D**–Transportome.
(XLSX)

S2 File Lists if high-weight SVM features. Lists for each input model type and each rhizosphere ecological niche are given on separate tables: **Table A**, Enzyme Function Profile–Biocontrol, Biofilm, Plant Pathogen, and Plant Growth; **Table B**, Metabolomic–Biocontrol, Biofilm, Plant Pathogen, and Plant Growth; **Table C**, Secondary Metabolism–Biocontrol, Biofilm, Plant Pathogen, and Plant Growth; **Table D**, Transportomic–Biocontrol, Biofilm, Plant Pathogen, and Plant Growth. Each table is a model output feature type. Within each table, rows are model output features and columns are model output type. A ‘1’ indicates that a model output feature is high-weight by SVM, ‘0’ otherwise.
(XLSX)
